# The Impact of Behavioral Lifestyle Intervention on Inflammatory Cytokines in Older Adults Living With Type 2 Diabetes: A Feasibility Study

**DOI:** 10.1177/00469580241248126

**Published:** 2024-04-20

**Authors:** Rozmin Jiwani, Monica Serra, Sara Espinoza, Andrea Berndt, Darpan Patel

**Affiliations:** 1 University of Texas Health Science Center at San Antonio, TX, USA; 2 South Texas Veterans Health Care System, San Antonio, TX, USA; 3 Cedars-Sinai Medical Center; 4 The University of Texas Medical Branch, Galveston, TX, USA

**Keywords:** behavioral lifestyle interventions, Inflammatory cytokines, frailty, older adults, type 2 diabetes, overweight/Obesity, feasibility study

## Abstract

**Objective:**

This study investigates the effects of a behavioral lifestyle intervention on inflammatory cytokines and frailty in older adults (≥ 65 years) with type 2 diabetes (T2D).

**Method:**

We conducted a single-arm, 6-month intervention supplemented with diet and activity self-monitoring technology. We assessed frailty using Fried criteria and quantified inflammatory cytokines (interleukin [IL]-2, IL-4, IL-6, IL-8, IL-10, granulocyte-macrophage colony-stimulating-factor [GM-CSF], interferon [IFN-γ], tumor necrosis factor [TNF-α]) using a multiplex assay. We used paired t-tests with significance at *P* < .05. We calculated the Spearman correlation and evaluated the relationship between frailty, BMI, and inflammatory cytokines.

**Results:**

Eighteen participants completed the study (mean ± SD: 71.5 ± 5.3 years; BMI: 34 ± 6 kg/m^2^). At baseline, we had 4 frail, 13 pre-frail, and 1 non-frail participant. At 6 months, we observed the therapeutic effects of the intervention on frailty score, BMI, IL-2, IFN-y, and GM-CSF.

**Discussion:**

The study highlights the importance of behavioral lifestyle intervention in improving inflammatory cytokines and frailty in older adults.

**What do we already know about this topic?**
The impact of behavioral lifestyle educational interventions (ie, promoting a healthy diet and physical activity) on the improvement of frailty, physical function, and body weight in individuals living with obesity and T2D.
**How does your research contribute to the field?**
The study results support the benefits of a behavioral lifestyle intervention in improving inflammatory cytokines, in addition to frailty, in individuals living with obesity and T2D.
**What are your research’s implications towards theory, practice, or policy?**
Recognizing the critical role of identifying effective lifestyle interventions to mitigate frailty and improve health span becomes imperative in promoting a better quality of life for older adults without reliance on pharmacological treatments.

## Introduction

Aging is a significant risk factor for chronic diseases, including type 2 diabetes (T2D) and cardiovascular diseases,^1,2^ often accompanied by frailty and low-grade inflammation (known as inflammaging).^3-5^ Frailty, a geriatric syndrome^6^ characterized by physical decline (loss of muscle mass, strength) and vulnerability, is associated with elevated inflammatory cytokines, such as interleukin (IL)-6 and tumor necrosis factor (TNF)-α.^1,7,8^ Type 2 diabetes and frailty independently pose significant risks, and their co-occurrence may exacerbate adverse outcomes.^9^ The unique vulnerabilities associated with frailty and type 2 diabetes underscore the need to understand the intricate interplay between these conditions and their collective influence on mortality. Further, preventing or improving frailty might be beneficial, in conjunction with glycemic control, for extending healthy life expectancy in persons with Type 2 diabetes (T2D).

Identifying behavioral lifestyle interventions (diet and regular physical activity) that improve healthspan and reduce frailty is crucial for promoting a better quality of life in the aging population.^10^ These interventions may offset the economic burdens of a burgeoning aging population and feasibly reduce frailty, ultimately leading to a better quality of life.^11-14^ We previously demonstrated the feasibility of behavioral lifestyle educational interventions (ie, promoting a healthy diet and physical activity), enhanced with mobile health technology, on the improvement of frailty, physical function, and body weight in individuals living with overweight/obesity and T2D.^12^ With this research, we expand on this work by evaluating whether the behavioral lifestyle educational interventions (ie, promoting a healthy diet and physical activity) also improve inflammatory cytokines and frailty. This study aimed to examine the effects of behavioral lifestyle educational intervention on inflammatory cytokines along with frailty.

## Methods

### Study Design and Recruitment

We recruited individuals living with overweight/obesity and T2D (≥65 years, BMI ≥25 kg/m^2^) with T2D for a single-arm 6-month (Jan-Dec 2020) behavioral lifestyle educational intervention that incorporated dietary and activity self-monitoring through mobile health technology. We previously published the study intervention and protocol details.^12^ We implemented a behavioral lifestyle educational intervention based on the Action for Health in Diabetes (Look AHEAD) Program.^15^ Participants received the intervention via ten sessions (60-90 min each) over 6 months. All group sessions focused on adherence to behavioral strategies, such as self-monitoring, goal setting, feedback, mindful eating, decreasing negative thoughts, social support, problem-solving, and relapse prevention. Participants received an individualized weight loss goal of 5% based on energy and fat intake based on their current weight, and physical activity goals to gradually increase to 175 min (about 3 h) per week by the end of the study under the Look AHEAD guidelines program.^15^ We provided the participants with a Fitbit wristband activity tracker and downloaded the companion Fitbit app on each participant’s smartphone. We instructed participants at the baseline visit and reminded them to self-monitor their diet and activity during every group session. We recorded body weight in every group session and reinforced intervention adherence.

**Ethical Considerations:** The Institutional Review Board at the University of Texas Health at San Antonio approved this study, and we registered it with clinicaltrials.gov (NCT04440449). Throughout the research process, we reminded participants of their voluntary involvement, mitigating any potential coercion. Furthermore, we implemented robust measures to protect the data gathered, utilizing a university password-locked server to adhere to general data protection regulations and maintain local ethical compliance.

#### Study subjects

In conducting this feasibility study, we selected a sample size of n = 20 for the organization of preliminary investigations in clinical and translation research. Anticipating a 40% attrition rate, we successfully recruited and enrolled 20 participants from the local communities.^16^ Individuals were eligible if they were aged ≥65 years, had a BMI ≥25 kg/m^2^ with self-reported T2D, and owned a smartphone. We excluded residents of a long-term care facility, those with a history of substance abuse in the past year, those with a history of severe psychiatric disorder, or those with cognitive impairment from this study. We held all study-related visits at the Biobehavioral Research Laboratory at University of Texas Health at San Antonio. All participants signed Institutional Review Board-approved informed consent forms.

### Procedures

As previously reported, Study assessments occurred at baseline and 6 months.^12^ After informed consent, we collected medical history, physical assessment, self-reported medical, surgical, and medication history, vital signs (heart rate, blood pressure), and anthropometric measurements (waist circumference [inches], height [inches], weight [pounds]). We calculated body mass index as weight (kg)/height (m^2^). We classified frailty status using the Fried phenotype criteria (ie, grip strength, walking speed, self-reported exhaustion, physical activity, and unintentional weight loss).^6,17^ Any intentional weight loss that may have occurred due to the lifestyle intervention would not meet the criteria for frailty-related weight loss, as we defined frailty-related weight loss as unintentional. We calculated the frailty score as the number (0-5) of frailty characteristics present and categorized those with ≥3 of these 5 characteristics as frail; those with 1 or 2 categorized as pre-frail, and those with none are categorized as non-frail.^6^ Blood was collected at the baseline and end of the study from participants via venipuncture in the antecubital space of the preferred arm. Plasma samples were obtained after speed centrifugation for 10 min at 2000 rpm and immediately stored at −80°C. We used a commercially available multiplex kit following the manufacturer’s recommended protocol (Millipore Sigma, Burlington, MA, USA) to quantify plasma concentrations of inflammatory cytokines (interleukin [IL]-2, IL-4, IL-6, IL-8, IL-10, granulocyte-macrophage colony-stimulating-factor [GM-CSF], interferon [IFN-γ], tumor necrosis factor [TNF-α]). We recorded the results in pg/ml.

### Statistical Methods

We used descriptive statistics, and all inflammatory cytokines were found to be right-skewed (ie, values of 1.00 or higher). Therefore, Wilcoxon signed-rank tests were used to examine changes in values pre- and post-intervention. We used Spearman correlation coefficients to assess the relationships between frailty score and BMI with inflammatory cytokines. We presented the data as means ± standard deviations, frequency, and percentage with *P* < .05 indicating statistical significance. Data were analyzed using SPSS (IBM, Version 28, Armonk, New York).

## Results

### Participant Characteristics

Two participants withdrew from the study because they were no longer interested in participating. Thus, we included the 18 participants who completed the study in the analyses. Participants were 56% female, 50% Hispanic, and the other half were non-Hispanic White, and on average age (71.5 ± 5.3 years) living with obesity (BMI: 33.7 ± 6.3 kg/m^2^). The parent study contains a detailed description of the participants’ characteristics, including a CONSORT diagram.^12^

### Therapeutic Effects of Behavioral Lifestyle Interventions on Frailty Score and BMI

We compared participants’ Fried frailty scores at baseline and the end of the study to determine if overall frailty scores improved during the study. At baseline, based on Fried criteria, we categorized 4 participants as frail, 13 as pre-frail, and 1 as non-frail. At 6 months, we categorized 2 participants as frail, 9 as pre-frail, and 7 as non-frail. Of the 18 participants, 8 (44%) demonstrated improvements in their frailty scores (2 changed from frail to pre-frail; 6 changed from pre-frail to not frail, respectively). Among the 8 participants with improved frailty scores, 7 (88%) exhibited intentional weight loss from baseline to the end of the study (average weight loss = 2.01 kg, range 0.20-3.80 loss in kg/avg percent weight loss = 6.16 SD = 3.89). The mean frailty score changed from 1.61 ± 1.15 to 0.94 ± 0.94 (−42%, *P* = .02). Participants BMI changed from 33.66 ± 6.28 to 32.51 ± 6.40 (−3%, *P* = .002).

### Therapeutic Effects of Behavioral Lifestyle Interventions on Inflammatory Cytokines

Table 1 presents the effect of the study intervention on inflammatory cytokines. At baseline, average values for all measured inflammatory cytokines were within normal ranges. At 6 months, significant reductions were observed for IL-2 (−33%; *P* = .04), IFN-γ (-36%; *P* = .03), and GM-CSF (−43%; *P* = .02).

**Table 1.** Effects of Study Intervention on Inflammatory Cytokines (Data in pg/ml).

	Reference mean values	Baseline mean (SD)	End of study mean (SD)	*P*-value
IL-2	12.1	3.08 ± 2.05	2.04 ± 0.52	*.04
IL-4	4.2	3.61 ± 3.27	2.53 ± 2.69	.16
IL-6	4.6	3.22 ± 3.25	2.79 ± 1.76	.95
IL-8	7.3	2.42 ± 1.63	2.15 ± 1.28	.64
IL-10	26.3	18.89 ± 15.85	17.58 ± 16.00	.83
IFN-γ	34.5	1.47 ± 1.32	0.92 ± 0.33	*.03
TNF-α	82.4	22.51 ± 13.63	19.36 ± 9.91	.45
GM-CSF	31.8	25.66 ± 23.98	14.68 ± 3.99	*.02

* p < .05

We examined the inflammatory markers of the 7 participants with improved frailty scores and intentional weight loss. Of these 7 participants, 6 exhibited reduced GM-CSF from baseline to the end of the study (avg GM-CSF = 18.39, range = 1.41–71.02/avg percent GM-CSF reduction = 46.80, range = 10.9-97.2) with lower baseline GM-CSF scores correlated to higher reductions at the end of the study (*r*_s_ = −.72, *P* = .11). Five of the seven participants exhibited unexpected changes to TNF-α scores. Specifically, higher baseline TNF-α scores correlated with higher increases by the end of the study TNF-α scores (*r*_s_ = −.82, *P* = .02).

### Relationship Between Frailty Scores and BMI with Inflammatory Cytokines

At baseline, we found a significant association between BMI and IL-6 (*r* = 0.49, *P* = .05). Following the intervention, we did not observe any other association between frailty score or BMI with inflammatory cytokines.

## Discussion

The study results support the hypothesis that a behavioral lifestyle intervention can improve inflammatory cytokine levels and frailty in individuals living with overweight/obesity and T2D. Our study supports previous findings indicating that inflammatory cytokines are an important pathophysiological signature associated with frailty.^3,5,18^

Our study expands on previous studies that have reported improvement in physical function, frailty, and reduction in inflammation using behavioral lifestyle interventions (ie, diet and physical activity).^19-21^ A review article investigated the effects of lifestyle interventions and reported improvement in proinflammatory cytokines, such as TNF-α and IL-6 (important mediators of inflammaging).^22^ Our results support this data by showing an association between intentional weight loss prescribed through our behavioral intervention and significant improvements in several inflammatory cytokines and frailty. Specifically, we observed that a 4% decrease in body weight was enough to illicit significant reductions in IL-2, IFN-γ, and GM-CSF. Similar to Look AHEAD study findings, we found no significant improvements for IL-8, IL-10, and TNF-α after lifestyle interventions.^23^ We also observed improvements in IFN-γ and GM-CSF concentrations, previously reported to exacerbate several inflammatory diseases associated with aging (eg, rheumatoid arthritis).^24^

The behavioral intervention implemented in our study educated participants on physical fitness and how to incorporate physical activity and a healthy diet into their daily routines. The variability in cytokine response to behavioral and lifestyle interventions is not unique to our study. Short-term, mild to moderate exercise training also reduced anti-inflammatory cytokines in individuals living with overweight/obesity and T2D.^25^ Continued research is warranted to fully understand the role of lifestyle modifications, including physical activity and diet, tailored to promote weight loss in persons living with overweight/obesity T2D and its relation to proinflammatory and anti-inflammatory cytokines balance.

Several strengths and limitations associated with this study need consideration when interpreting the results. Strengths of this study include the high retention rate (90%), good adherence to the study interventions as evidenced by the activity tracker data,^12,26^ and successful improvements in BMI, frailty, and inflammatory cytokines at 6 months. Further, we performed our study among community-dwelling racially diverse individuals living overweight/obesity, with a mean age of 71.5 years, mean BMI of 33.7 kg/m^2^, and most with pre-frail/frail range frailty scores that urged the importance of early intervention to maintain healthspan and quality of life in this group. Limitations of this study include the non-experimental, single-arm, non-randomized study design. Second, this study’s small sample size could affect the generalizability and interpretation of these results. The disrupted setting consistency due to the transfer of group sessions from in-person to virtual (WebEx) after the COVID-19 outbreak could have influenced results.^12,26^ Finally, we did not prescribe a specific diet or exercise program in our study. Therefore, the foods participants consumed, or their sedentary lifestyle combined with COVID-19 stress could have impacted inflammatory markers. The intervention’s effectiveness in reducing proinflammatory cytokines and frailty underscores its potential as a non-pharmacological therapy for this population. However, we need further research with larger sample sizes and controlled designs to confirm and expand these findings and explore the specific impact of lifestyle modifications on proinflammatory and anti-inflammatory cytokines balance.

## Conclusion

This study highlights the importance of behavioral lifestyle interventions in the context of individuals living with overweight/obesity and T2D, focusing on the improvement of inflammatory cytokines and frailty. Importantly, existing research suggests that achieving a moderate weight loss of 3% to 5% can yield substantial health benefits, contributing significantly to overall well-being. This range of weight loss has been associated with enhanced glycemic control and reduced inflammation, making it a crucial aspect to consider in the broader implications of our findings. These findings contribute valuable insights into the potential health benefits from behavioral lifestyle interventions in this vulnerable population. Recognizing the critical role of identifying effective lifestyle interventions to mitigate frailty and improve healthspan becomes imperative in promoting a better quality of life for older adults without reliance on pharmacological treatments. Future larger-scale research studies should explore implementing such interventions in clinical practice to benefit patients with T2D and comorbidities.
